# The Territorial Medical Officer's Examinations

**Published:** 1911-05-06

**Authors:** 


### The Royal Army Medical Corps Section.

### THE TERRITORIAL MEDICAL OFFICER'S EXAMINATIONS.

There has recently been issued by the military authorities a return which gives the results of the last examinations held for Territorial Officers, both combatant and medical. It is with the latter only that we are chiefly concerned. We observe that out of sixty candidates thirty-nine have passed and twenty-one failed in one or more subjects, a rather large percentage of non-successes. Before making any observations on this point it may be as well to mention in detail the different examinations the Territorial medical officer after joining has to pass so as to render him eligible for promotion to the higher ranks.

### The Examinations.

The first examination is for captain and must be got through within two years after being gazetted. It consists of examination A (practical and oral) and examination B (written). The scope of the former is comprised in R.A.M.C. Training Part II. (omitting chapter I.), and includes a knowledge of drill and corps work, such as first aid and the various means of moving the wounded, while the latter takes in (1) military law, (2) organisation, administration, and equipment, and (3) sanitation and other duties in camp, barracks, and the field. The books to be used in preparing for these subjects with the special chapters and paragraphs to be studied are given in Appendix IV. of the Territorial Regulations, as are also the points in organisation, administration, and equipment, of which a detailed knowledge will be expected. The printed papers are set at the War Office by the Director of Military Training, and examinations are held twice yearly at different centres. After attaining the rank of captain no examination for promotion to the rank of major is required from Territorial medical officers. What is substituted for it is an eight working days' course at a R.A.M.C. (T.F.) school or at a selected military institution, there being however required, in addition, a riding certificate, which must be to the effect that the candidate can ride sufficiently well to perform the duties of a mounted officer. For promotion from the rank of major to that of lieutenant-colonel it is necessary to pass examination D, which is an entirely written one. To be successful an officer must obtain 0.6 of the marks allotted to each of the subjects, which are three in number, namely: (1) The medical organisation of the Territorial Force in peace and war; (2) the sanitation of towns, camps, and all places likely to be occupied by Territorial Force troops in peace and war, as well as epidemiology and management of epidemics; and (3) "The laws and customs of war on land" so far as they relate to the sick and wounded.

### Subjects.

As regards the subject-matter of the different examinations mentioned above, no fault can be found with it. To the lot of the Territorial medical officer there fall both administrative as well as professional duties, and it is very necessary that he should have a knowledge of how to conduct the proceedings of a court-martial, of the rules relating to courts of inquiry, and of the laws of evidence. He should also be acquainted with the regulations for the administration, organisation, and equipment of the Territorial Force, so that he may deal with all the many subjects involved, such as the issue of camp equipment for Territorial camps, the requisitioning of supplies in the field, the organisation and lines of communication in war, the occasions on which pay and allowances may be drawn by officers, non-commissioned officers, and men of the Territorial Force, the necessary steps for the mobilisation of his unit, and all similar matters. It is also very necessary that he should be tested in his knowledge generally of sanitation, so that he may show he is acquainted with the many details included under it, such as the location and management of kitchens in camp, the disposal of slop water and refuse, the sterilisation of water, the inspection of meat and food supplies in a camp, and the arrangements called for should there be a prolonged occupation of a camp. To be well versed, too, in the laws and customs of war on land so far as they relate to the sick and wounded is a very necessary qualification, and we hold that its inclusion in the subjects for examination is in every way justified.

### Some Conclusions.

In view of the large percentage of non-successes in the examinations held recently, it is incumbent on the military authorities to satisfy themselves that this examination test for efficiency is fairly employed and is accompanied by proper facilities for instruction both regimentally and otherwise. If it is the case that success in these examinations can only be attained under conditions that involve for the Territorial medical officer personal worry and inconvenience, to say nothing of monetary outlay, then he is not having in this matter fair treatment. It is not sufficient to furnish the list of books to be read in connection with each subject. There should also be provided that skilled assistance that is necessary for the thorough understanding of much that is technical and not easily grasped by the civilian mind unaccustomed as it is to many of the problems presented to it. It is wise to bear in mind, too, that examinations have their faults as well as their advantages, and that failures to reach the standard laid down might be due in some cases to want of intelligence and inattention to study, but in others to defective facilities for instruction or to the fault of the examiners. In the training of Territorial medical officers, just as in those of other ranks, what should be aimed at is to ensure not mere theoretical excellence but true practical capacity in the field. This can only be obtained by practical training, and this is undoubtedly what the Territorial officer and soldier chiefly needs.

